# Efficacy and safety of toripalimab in the treatment of nasopharyngeal carcinoma: a meta-analysis of single-arm trials

**DOI:** 10.1186/s12865-025-00744-1

**Published:** 2025-08-25

**Authors:** Huiping Zhang, Mengyuan Liu, Le Yan, Rongqiu Hu, Lingtong Dai

**Affiliations:** 1 School of Medical and Life Sciences, Chengdu University of Traditional Chinese Medicine, No. 37, Twelfth Bridge Road, Chengdu City, Sichuan, China; 2 Qingdao Hospital, University of Health and Rehabilitation Sciences (Qingdao Municipal Hospital), Qingdao, China; 3 Sichuan Vocational College of Commerce, Chengdu, China; 4 Affiliated Hospital of Panzhihua University, No.25 Taoyuan Street, Panzhihua City, 636500 Sichuan China

**Keywords:** Toripalimab, Nasopharyngeal carcinoma, Efficacy, Adverse events, Meta-analysis

## Abstract

**Background:**

Nasopharyngeal carcinoma (NPC) is common in East Asia, and treatment options are limited. Toripalimab, a PD-1 inhibitor, has potential in NPC therapy.

**Objective:**

This meta-analysis aimed to evaluate the efficacy and safety of toripalimab in the treatment of NPC.

**Methods:**

A systematic search of PubMed, Embase, the Cochrane Library and Web of Science was conducted to identify studies assessing toripalimab in NPC. The search time ranged from the establishment of the database to January 10, 2025. R4.4.1 software was used for data analysis.

**Results:**

Nine papers (with a total of 801 patients) were included, of which 2 were randomized controlled studies and 7 were single-arm studies. Meta-analysis results suggest that after treatment with toripalimab, the ORR of NPC was 77% (95% CI 55%-90%)], DCR was 93% (95% CI 81%-100%), 1-year OS was 81% (95% CI 43%-100%), 2-year OS was 68% (95% CI 0%-100%), 3-year OS was 75% (95% CI 42%-97%), 1-year PFS was 81% (95% CI 62%-91%), 2-year PFS was 56% (95% CI 26%- 82%), 3-year PFS was 25% (95% CI 7%-60%), the rate of adverse events was 98% (95% CI: 90%-100%), and the rate of grade ≥ 3 was 76% (95% CI: 52%-94%).

**Conclusions:**

These results suggest that toripalimab has better efficacy in the treatment of NPC, especially in terms of disease control and survival. However, it was also accompanied by a high incidence of adverse events, especially grade ≥ 3 adverse events. Because there was no control group in this study, caution is needed when interpreting the results.

**Supplementary Information:**

The online version contains supplementary material available at 10.1186/s12865-025-00744-1.

## Background

Nasopharyngeal carcinoma (NPC) is a malignant tumour originating from nasopharyngeal epithelial cells with a high endemic distribution, especially in Southeast Asia [1, 2]. According to the World Health Organization (WHO), the annual incidence rate of NPC is approximately 1/100,000 people globally, but the incidence rate can reach 20/100,000 people in southern China, especially in Guangdong, Guangxi and Hainan, representing high incidence areas of NPC [3]. Although radiotherapy and chemotherapy are the standard treatments for nasopharyngeal cancer, traditional treatments have limited effectiveness and poor prognosis for patients with locally advanced or recurrent metastases [4, 5]. According to the Chinese Tumor Registry, the five-year survival rate of patients with nasopharyngeal cancer is approximately 60% to 70%; however, for patients with advanced stages, the survival rate is low [6]. Therefore, there is an urgent need to develop new therapeutic options to improve the survival rate and quality of life of patients.

In recent years, significant progress has been made in the use of immunotherapy in cancer treatment, especially the use of immune checkpoint inhibitors (anti-PD-1/PD-L1 antibodies), which have been shown to exhibit promising efficacy in the treatment of a wide range of cancers [7]. PD-1 is an inhibitory immune receptor, and in the tumour microenvironment, tumour cells, through the binding of PD-1 to its ligands PD-L1 and PD-L2, inhibit the immune response of T cells and evade immune system surveillance [8, 9]. An anti-PD-1 monoclonal antibody restores T-cell function and enhances the body's immune response by blocking the binding of PD-1 to PD-L1, thus effectively inhibiting tumour growth [10].

Toripalimab, an anti-PD-1 monoclonal antibody, has achieved good clinical results in the treatment of a variety of tumours, including nasopharyngeal cancer. In 2018, toripalimab received the first marketing approval in China for advanced or recurrent nasopharyngeal cancer [11]. Although the potential of toripalimab in the treatment of NPC was initially demonstrated, some unanswered questions remain. For example, the optimal dose of toripalimab, the duration of treatment, and its use in combination with other therapies still require further study [12]. In addition, although randomized controlled trials (RCTs) provide high-quality evidence, large-scale RCTs are relatively difficult to conduct because of the limited number of patients with nasopharyngeal cancer. Therefore, single-arm studies have emerged as effective methods for assessing the efficacy and safety of new drugs in this rare type of tumour. A meta-analysis of single-arm studies allows for the synthesis of data from different studies and supports more accurate conclusions.

The aim of this meta-analysis was to evaluate the efficacy and safety of toripalimab in the treatment of NPC and to systematically assess its clinical efficacy in patients with NPC by pooling data from relevant studies, including the objective response rate (ORR), disease control rate (DCR), progression-free survival (PFS), and overall survival (OS), as well as its safety profile. On the basis of the combination of existing single-arm studies and randomized controlled studies, a more comprehensive understanding of the role of toripalimab in the treatment of NPC can be obtained, and a scientific basis for clinical practice can be provided.

## Methods

The PRISMA checklist was followed for this study, and this study was preregistered on the Prospero platform with registration number CRD420250650943 (https://www.crd.york.ac.uk/PROSPERO/view/CRD420250650943).

### Literature search

A systematic search of PubMed, Embase, the Cochrane Library and Web of Science was conducted from database creation to 10 January 2025. The search terms used were toripalimab and NPC, and the specific search strategy is described in Supplementary Material Table S1.

### Inclusion and exclusion criteria

The inclusion criteria were as follows: the study was a single-arm clinical study or an RCT, the efficacy and safety of toripalimab in the treatment of NPC were assessed, the study was conducted in patients with a definitive diagnosis of nasopharyngeal cancer (which can be advanced, metastatic, locally recurrent or inoperable), the primary outcomes included ORR, DCR, PFS and OS, and the secondary outcomes included adverse events (AEs) of any grade and serious adverse events (SAEs). In addition, there were no language restrictions, and studies could be journal articles or conference abstracts.

The exclusion criteria included studies with concurrent use of other immunotherapies or targeted agents or studies that did not explicitly report toripalimab as a monotherapy agent, studies that lacked efficacy or safety data or did not report key outcomes (ORR, DCR, PFS, OS); animal experiments; in vitro studies; and preclinical studies. Only clinical trial data were valid. Duplicate published studies were also excluded to ensure data independence and reliability. Finally, studies on patients without nasopharyngeal cancer did not meet the inclusion criteria, and only studies in nasopharyngeal cancer patients were considered.

### Data extraction

Two authors independently screened the literature using EndNote 21 software according to the inclusion and exclusion criteria. If the two authors were in dispute, they could discuss the results with each other or seek the third author's help. The main data extracted for this study included basic study characteristics (first author, year of publication, country, and experimental design), demographic characteristics (sample size, sex, age, tumour type, and TNM stage), dose, combination therapy, PD-L1 positivity, and site of metastasis. For articles with no available data and those for which the full text was not available, an attempt was first made to obtained this information by contacting the corresponding author. If the author was not available, the article was excluded.

### Assessment of quality

For the RCTs, the risk of bias of the studies was assessed using the Cochrane Risk of Bias Tool 2.0 (RoB2) [13]. RoB2 is applied by two independent investigators to assess potential bias in several domains, including the randomization process, deviation from the intended intervention, missing outcome data, selection of outcome measures, and reporting of results. If the two investigators did not agree on the assessment, a third investigator was consulted to reach a consensus. Studies were categorized as having a low, medium or high risk of bias on the basis of the evaluation.

For single-arm studies, the quality of the studies was assessed using the Newcastle–Ottawa Scale (NOS) [14]. The NOS scores were based on three main categories: selectivity, comparability and outcome. Assessment scores of 0–3, 4–6 and 7–9 indicate poor, fair and good study quality, respectively. Any differences in quality assessment between researchers were resolved by consensus.

### Statistical analysis

Statistical analysis for this study was performed using the ‘meta’ package in R.4.4.1 (The R Foundation for Statistical Computing). This study used combined proportions and 95% CIs and tests for I2 values or the Q statistic to test for heterogeneity. I2 values of 0%, 25%, 50% and 75% represent no, low, medium and high heterogeneity, respectively. When I2 values were ≥ 50%, sensitivity analyses were performed to explore potential sources of heterogeneity. If heterogeneity was < 50%, a fixed-effects model was used. Publication bias was assessed using funnel plots.

## Results

### Literature search results

**Fig. 1:**
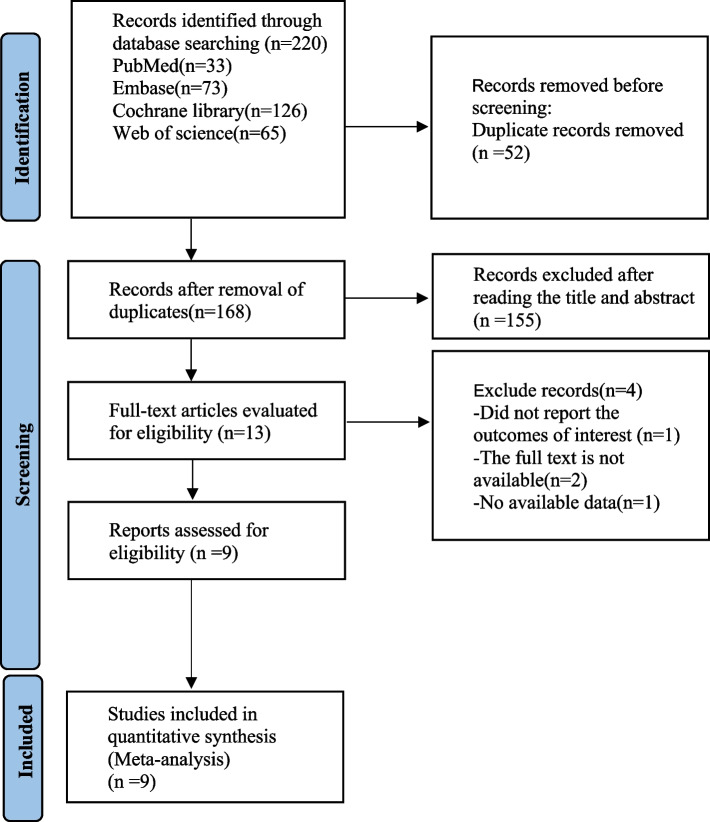
Literature search flowchart

As shown in Fig. 1, a search of PubMed (*n* = 33), Embase (*n* = 73), the Cochrane Library (*n* = 126), and Web of Science (*n* = 65) yielded a total of 220 articles. The number of articles was reduced by removing duplicates (*n* = 52), removing titles and abstracts (*n* = 155), and reading the full texts (*n* = 4). In total, 9 articles were ultimately included.

### Basic characteristics of the studies

In the present study, 9 papers [15–23] were included, of which 2 were randomized controlled studies [18, 23] and 7 were single-arm studies [15–17, 19–22]. A total of 801 patients were included, with patient ages ranging from 46.455 years. Of these papers, 2 articles [15, 18] were focused on locally advanced disease, and 7 [16, 17, 19–23] were focused on recurrent metastatic NPC with metastatic sites in the liver, bone, or lungs. The specific basic features are shown in Table 1.

**Table 1:** Table of basic characteristics

Randomized controlled study											
Study	Year	Country	Sample size	Gender(M/F)	Mean age(years)	TNM stage	Types	Dose	Combination Therapy	PD-L1 positive	Metastatic site
Liu	2024	China	T:100 P:50	115/35	T:47 P:46.5	III-IVA	Locally advanced	240mg	chemoradiotherapy	T:72 P:34	NR
Mai	2023	China	T:146 P:143	240/49	T:46 P:51	NR	Recurrent/Metastatic	240mg	Chemotherapy	T:109 P:109	Liver, bone, lungs
Single arm study											
Study	Year	Country	Sample size	Gender(M/F)	Mean age(years)	TNM stage	Types	240mg	Combination Therapy	PD-L1 positive	Metastatic site
Cao	2024	China	23	15/8	52	II-IVA	Locally advanced	240mg	Chemotherapy	NR	NR
Chen	2023	China	22	15/7	54.5	NR	Recurrent/Metastatic	240mg	chemoradiotherapy	NR	Liver, bone, lungs
Hua	2021	China	25	18/7	49	II-IVA	Recurrent/Metastatic	240mg	radiotherapy	NR	NR
Wang	2021	China	190	158/32	46.4	III-IVB	Recurrent/Metastatic	3mg/kg	chemoradiotherapy	48	NR
You	2022	China	41	30/11	46	NR	Recurrent/Metastatic	240mg	Chemotherapy + VEGFR	NR	Liver, bone, lungs
Zhang	2024	China	40	34/6	46	NR	Recurrent/Metastatic	240mg	Chemotherapy + VEGFR	NR	Liver, bone, lungs
Zou	2024	China	21	15/6	55	NR	Recurrent/Metastatic	240mg	Chemotherapy	NR	Liver, bone, lungs

*M/F* male/female, *NR* not reported, *VGFR* anti-vascular endothelial growth factor

### Quality evaluation results

For the two randomized controlled studies, the randomization methodology was clearly explained and therefore assessed as low risk, as described in Supplementary Material Figure S1. For the single-arm studies, the NOS scores ranged from 5 to 8, which is of moderate quality, as described in Supplementary Material Table S2.

### Meta-analysis results

#### Objective response rate (ORR)

**Fig. 2:**
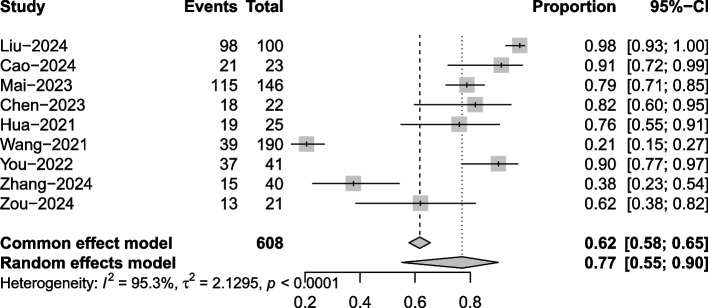
Objective response rate meta-analysis forest plot

Nine articles mentioned the ORR. Due to significant heterogeneity (*I*^2^ = 95.3%, *P* = 0.0001), a random effects model was used for the analysis. The results of the analysis (Fig. 2) suggested that after treatment with toripalimab, the ORR of NPC was 77% (95% CI 55%−90%).

Subgroup analysis of the combination therapy results (Supplementary Material Figure S2) suggested that the ORR of NPC was 79% (95% CI 50%−94%). For toripalimab combined with chemoradiotherapy and vascular endothelial growth factor (VEGFR) inhibitors, the ORR of NPC was 70% (95% CI 25%−94%). Based on the type of NPC, the analysis results (Supplementary Material Figure S3) suggested that for locally advanced NPC, after toripalimab treatment, the ORR was 97% (95% CI 92%−99%). For metastatic/recurrent NPC, after toripalimab treatment, the ORR was 66% (95% CI 44%−88%).

Sensitivity analysis was performed with one-by-one exclusion, and the results of the analysis (Supplementary Material Figure S4) suggested minimal change in heterogeneity after the exclusion of each paper individually.

#### Disease control rate (DCR)

**Fig. 3:**
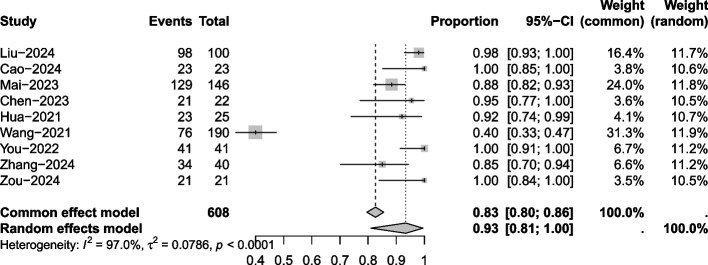
Disease control rate meta-analysis forest plot

Nine articles mentioned DCR. Due to significant heterogeneity (*I*^2^ = 97%, *P* = 0.0001), a random effects model was used for the analysis. The results of the analysis (Fig. 3) suggested that after treatment with toripalimab, the DCR of NPC was 93% (95% CI 81%−100%).

Subgroup analysis of the combination therapy results (Supplementary Material Figure S5) suggested that the DCR of NPC was 93% (95% CI 73%−100%). For toripalimab combined with chemoradiotherapy and vascular endothelial growth factor (VEGFR) inhibitors, the DCR of NPC was 96% (95% CI 69%−100%). In terms of the type of NPC, the analysis results (Supplementary Material Figure S6) suggested that for locally advanced NPC, after toripalimab treatment, the DCR was 99% (95% CI 95%−100%). For metastatic/recurrent NPC, after toripalimab treatment, the DCR was 91% (95% CI 73%−99%).

Sensitivity analysis was performed with one-by-one exclusion, and the results of the analysis (Supplementary Material Figure S7) suggested minimal change in heterogeneity after excluding the papers one-by-one.

#### Overall survival (OS)

**Fig. 4:**
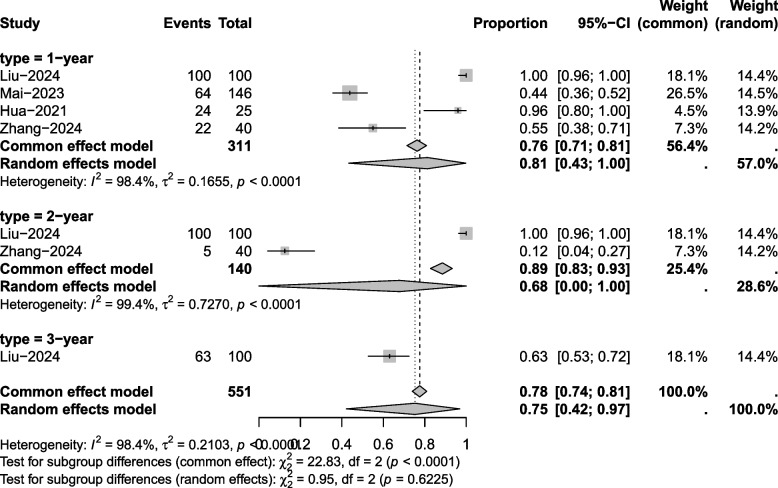
Overall survival meta-analysis forest plot

Four articles mentioned OS. Significant heterogeneity was noted (*I*^2^ = 98.4%, *P* = 0.001), so a random-effects model was employed. The results of the analysis (Fig. 4) revealed a 1-year OS of 81% (95% CI 43%−100%), 2-year OS of 68% (95% CI 0%- 100%), and 3-year OS of 75% (95% CI (42%−97%).

#### Progression-free survival (PFS)

**Fig. 5:**
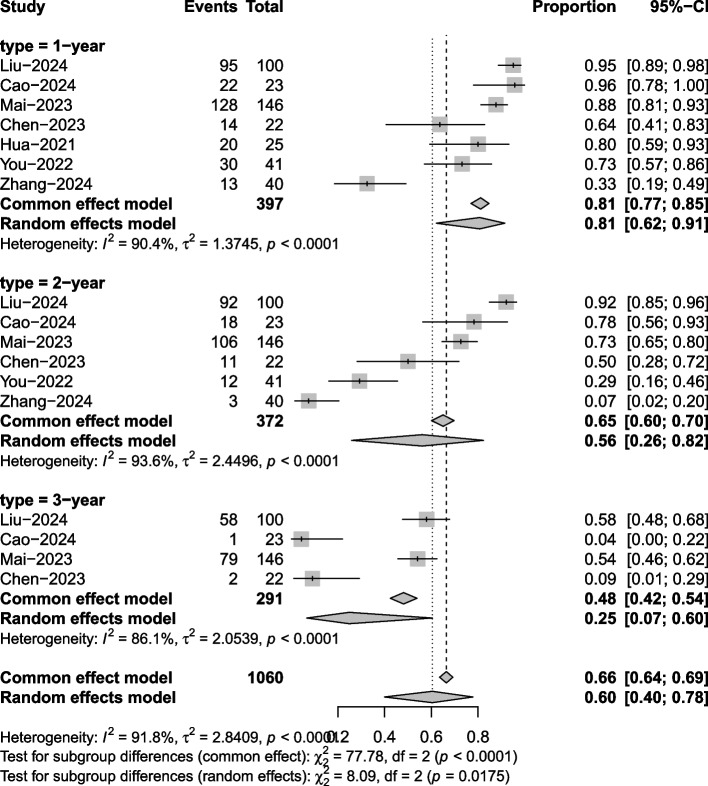
Progression-free survival meta-analysis forest plot

Seven articles mentioned PFS. Significant heterogeneity was noted (I^2^ = 91.8%, P = 0.001), so a random effects model was used. The results of the analysis (Fig. 5) revealed a 1-year PFS of 81% (95% CI 62%−91%), 2-year PFS of 56% (95% CI 26%- 82%), and 3-year PFS of 25% (95% CI 7%−60%).

#### Adverse events

Table 2 shows the incidence rates of various adverse events. For all adverse events, the proportion was 98% (95% CI: 90%−100%). For grade III and above adverse events, it was 76% (95% CI: 52%−94%). The incidence of anaemia was 61% (95% CI: 30%−88%), with 9% (95% CI: 1%−22%) of patients experiencing grade III or above anaemia. Diarrhoea occurred in 20% (95% CI: 11%−31%) of the patients, with grade III diarrhoea or above occurring in 1% (95% CI: 0%−2%) of the patients. The incidence of leukopenia was 46% (95% CI: 25%−68%), with the incidence of patients with grade III and above leukopenia noted in 10% (95% CI: 1%−25%). Nausea was present in 76% (95% CI: 57%−88%) of the cases, with grade III and above noted in 2% of patients (95% CI: 0%−3%). Rash occurred in 19% of the patients (95% CI: 11%−31%), with grade III and above noted in 1% of patients (95% CI: 0%−4%). Thrombocytopenia was observed in 16% (95% CI: 7%−34%) of the patients, with grade III thrombocytopenia and above noted in 4% of patients (95% CI: 0%−12%).

**Table 2:** Meta-analysis results of adverse events

Any grade					Grade ≥ 3				
Adverse events	No of study	I^2^(%)	proportion (95%CI)	*P*	Adverse events	No of study	I^2^(%)	proportion (95%CI)	*P*
Any	6	95.6	0.98(0.90, 1.00)	< 0.0001	Any	6	91.7	0.76(0.52, 0.94)	< 0.0001
Anemia	8	84.8	0.61(0.30, 0.88)	< 0.0001	Anemia	8	95.9	0.09(0.01 0.22)	< 0.0001
Diarrhea	8	84.8	0.20(0.11, 0.31)	< 0.0001	Diarrhea	8	26.1	0.01(0.00, 0.02)	< 0.0001
Leukopenia	9	98.0	0.46(0.25, 0.68)	< 0.0001	Leukopenia	9	97.5	0.10(0.01, 0.25)	< 0.0001
Nausea	7	84.7	0.76(0.57, 0.88)	< 0.0001	Nausea	7	0	0.02(0.00 0.03)	< 0.0001
Rash	9	85.7	0.19(0.11, 0.31)	< 0.0001	Rash	9	68.3	0.01(0.00, 0.04)	0.0014
Thrombocytopenia	8	90.8	0.16(0.07, 0.34)	< 0.0001	Thrombocytopenia	8	92.0	0.04(0.00, 0.12)	< 0.0001

#### Publication bias

In the present study, funnel plots and Egger tests were used to detect publication bias, and the results of the analyses were not symmetrical on either side (Supplementary Material Figures S8-S11), suggesting a low probability of publication bias. Egger test results (Supplementary materials Table S3) revealed that the *P* values were all > 0.05, indicating that the possibility of publication bias was low.

## Discussion

Compared with previous meta-analyses, previous studies [24, 25] have explored the efficacy and safety of all PD-1/PD-L1 agents for NPC. However, owing to differences in drug types, administration methods, and dosages, these studies exhibited greater heterogeneity. Therefore, this study focused on the efficacy of toripalimab for the treatment of NPC and included a larger number of studies to identify new treatment options for patients with NPC.

This is the first study in which the efficacy and safety of toripalimab in NPC have been analysed using a single-arm meta-analysis, which evaluated the efficacy and safety of toripalimab in the treatment of NPC. The results of this study revealed that toripalimab was significantly effective in improving the ORR and DCR of patients, especially when combined with chemoradiotherapy and VEGFR inhibition. Among patients with different types of NPC, patients with locally advanced NPC had better outcomes, whereas patients with metastasis or recurrence had relatively worse outcomes. In addition, toripalimab significantly improved patient survival, and PFS was also more favourable. Although on-treatment adverse events, such as anaemia, diarrhoea and leukopenia, are relatively common, most of them are manageable mild to moderate adverse events, and sensitivity analyses have shown robust results with some potential for clinical application.

This study’s subgroup analysis revealed that the ORR was 79% (95% CI: 50%−94%), and the DCR was 93% (95% CI: 73%−100%). However, when it was combined with chemoradiation and VEGFR inhibitors, the ORR was 70% (95% CI: 25%−94%), and the DCR was 96% (95% CI: 69%−100%). These results suggest that toripalimab not only improves treatment efficacy when combined with other treatment regimens but also performs better in terms of both ORR and DCR when combined with chemoradiotherapy. However, although the ORR remained at approximately 70% and the DCR was greater (96%) when this therapy was combined with VEGFR inhibitory therapy. The wide range of 95% CIs suggests that the efficacy of this combination therapy still exhibits considerable uncertainty. This discrepancy may be related to the effects of different treatment combinations on the immune microenvironment and patient tolerance to different treatment regimens [26, 27]. Chemotherapy and radiotherapy may potentiate the antitumour response of the immune system by inducing immunogenic cell death, which may have a synergistic effect with toripalimab [28]. On the other hand, VEGFR inhibitory therapy reduces the blood supply to the tumour mainly by blocking tumour angiogenesis, which may affect the tumour immune microenvironment and immune cell activity, thus affecting the efficacy of toripalimab [29, 30]. Therefore, although the combination of toripalimab and VEGFR inhibitory therapy has shown some efficacy, there may be additional complexities in the immune escape mechanisms and vascular remodelling of tumours, which may lead to greater variation in the efficacy of this combination in different patient groups.

According to the results of the analysis of different types of NPC patients, the ORR and DCR of patients with locally advanced NPC were 97% (95% CI: 92–99%) and 99% (95% CI: 95–100%), respectively, indicating that toripalimab has a very significant effect on these patients. For patients with metastatic/recurrent NPC, the ORR was 66% (95% CI: 44%−88%), and the DCR was 91% (95% CI: 73–99%), which was relatively low but still reflected the efficacy of toripalimab in these patients. This difference suggests that toripalimab has significant efficacy in the treatment of locally advanced nasopharyngeal cancer, especially when immunotherapy can play its full role. For metastatic or recurrent patients, the relatively low ORR may be related to the biological characteristics of the tumour and the immune escape mechanism [31]. Patients with locally advanced disease usually receive more focused and localized treatments (radiotherapy, chemotherapy), which may help improve the ability of the immune system to recognize and clear tumours, thus allowing immune checkpoint inhibitors such as toripalimab to work better [32, 33]. In contrast, tumours in patients with metastatic or recurrent disease usually exhibit more immune escape mechanisms, such as alterations in the tumour microenvironment and depletion of immune cells [4, 34]. This may result in immunotherapy being less effective in patients with metastatic or recurrent disease compared with patients with locally advanced disease. In addition, treatment options are more complex for patients with metastatic and recurrent NPC, who may have received multiple rounds of therapy and experienced more complex changes in their immune microenvironment, which may require a more personalized treatment regimen [35]. Therefore, although toripalimab has shown some efficacy in these patients, more clinical data are needed to optimize treatment strategies and improve treatment outcomes.

According to the results of this single-arm meta-analysis, although toripalimab showed some efficacy in the treatment of nasopharyngeal cancer, the high heterogeneity suggests that there may be significant differences between studies, affecting the reliability of their results. First, in the analysis of OS and PFS, despite some survival benefit, the heterogeneity of both OS and PFS was very high, at 98.4% and 91.8%, respectively. This finding suggests that differences between studies, such as study design, patient characteristics, and treatment protocols, may have an impact on the evaluation of treatment effectiveness and that OS and PFS are accompanied by wide confidence intervals, indicating a degree of imprecision in these estimates. This could be attributed to factors such as sample size variability, study design differences, and heterogeneity in patient characteristics across the included studies. Although these results provide valuable insight, further studies with larger and more homogenous patient populations are needed to improve the precision of these estimates. Therefore, further large-scale, multicentre clinical studies are warranted to verify the actual efficacy and safety of toripalimab in different populations. In addition, although toripalimab has shown potential in prolonging patient survival, side effects, especially anaemia and nausea, are relatively common. Although the overall incidence of anaemia is high, the incidence of anaemia of Grade III and above is low, which may indicate that the degree of anaemia is relatively mild or manageable in some patient groups [36]. Nausea is another common side effect. Although it occurs at a relatively high rate, most nausea symptoms are mild in severity. Although serious adverse events are rare, these side effects still require close monitoring and timely intervention in clinical treatment to reduce patient suffering and reduce the risk of complications.

In summary, toripalimab has shown some clinical value in the treatment of NPC. However, given its high heterogeneity and nonnegligible side effects, more multicentre, large-sample clinical trials are recommended to further evaluate its efficacy and safety and optimize patient management strategies.

### Limitations

The current study has several limitations. Most of the studies included were single-arm trials lacking control groups, which may lead to bias and affect the accuracy of efficacy assessments. Additionally, some studies had incomplete long-term follow-up data, with wide confidence intervals for OS and PFS, indicating insufficient follow-up or missing data, which could impact the reliability of the results. Variability in patient selection, treatment regimens, and follow-up periods across studies introduced heterogeneity, limiting the generalizability of the conclusions. Furthermore, publication bias could have influenced the findings, as studies with positive results may be more likely to be published. The small sample sizes in some studies may also have contributed to imprecision in the estimates. Finally, the heterogeneity introduced using combination therapies across the included studies may have influenced the overall results, making them less generalizable to different treatment settings.

## Conclusion

These results suggest that toripalimab has better efficacy in the treatment of NPC, especially in terms of disease control and survival. However, it was also accompanied by a high incidence of adverse events, especially grade ≥ 3 adverse events. Future studies could focus on balancing efficacy and safety, further optimizing treatment regimens, and exploring the potential of combination therapies to improve patient survival and reduce the incidence of adverse events.

## Supplementary Information

Supplementary Material 1

## Data Availability

The data used for the analyses are available upon reasonable request from the first author. The data sets supporting the conclusions of this article are included within the article and its additional files.
