# Supplementary material for: Efficacy and safety of toripalimab in the treatment of nasopharyngeal carcinoma: a meta-analysis of single-arm trials
Source: BMC Immunol. 2025 Aug 25;26:62. doi: 10.1186/s12865-025-00744-1 (PMC12379329; doi:10.1186/s12865-025-00744-1)
Supplement: Supplementary file 1 — Supplementary Material 1 [file 12865_2025_744_MOESM1_ESM.docx]

Table S1 search strategy

| PubMed | (("Nasopharyngeal Carcinoma"[Mesh]) OR ((((Nasopharyngeal Carcinoma [Title/Abstract]) OR (Carcinoma, Nasopharyngeal[Title/Abstract])) OR (Carcinomas, Nasopharyngeal[Title/Abstract])) OR (Nasopharyngeal Carcinomas[Title/Abstract]))) AND (toripalimab[Title/Abstract]) |
| --- | --- |
| Embase | \| #8 \| #6 AND #7 \| \| --- \| --- \| \| #7 \| 'toripalimab':ab,ti \| \| #6 \| #1 OR #2 OR #3 OR #4 OR #5 \| \| #5 \| 'nasopharyngeal carcinomas':ab,ti \| \| #4 \| 'carcinomas, nasopharyngeal':ab,ti \| \| #3 \| 'carcinoma, nasopharyngeal':ab,ti \| \| #2 \| 'nasopharynx carcinoma':ab,ti \| \| #1 \| 'Nasopharynx carcinoma'/exp \| |
| Cochrane library | #1 MeSH descriptor: [Nasopharyngeal Carcinoma] explode all trees  #2 (Nasopharyngeal Carcinoma):ti,ab,kw OR (Carcinoma, Nasopharyngeal):ti,ab,kw OR (Carcinomas, Nasopharyngeal):ti,ab,kw OR (Nasopharyngeal Carcinomas):ti,ab,kw  #3 #1or#2  #4 (toripalimab):ti,ab,kw  #5 #3and#4 |
| Web of science | 1 TS=(Nasopharyngeal Carcinoma) OR TS=(Carcinoma, Nasopharyngeal) OR TS=(Carcinomas, Nasopharyngeal) OR TS=(Nasopharyngeal Carcinomas)  2 TS=(toripalimab)  3 #1 AND #2 |

Table S2 NOS scores

| Newcastle-Ottawa Scale (NOS) for Single arm studies | | | | |
| --- | --- | --- | --- | --- |
| Study | Selection | Comparability | Exposure | Total score |
| Cao2024 | ******* | ****** | ****** | 7 |
| Chen2023 | ****** | ****** | ****** | 6 |
| Hua2021 | ******* | ***** | ****** | 5 |
| Wang2021 | ******* | ****** | ******* | 8 |
| You2022 | ******* | ****** | ****** | 7 |
| Zhang2024 | ****** | ****** | ******* | 7 |
| Zou2024 | ******* | ****** | ******* | 8 |

*: one score

Table S3 Egger test

| Outcomes | Egger Test |
| --- | --- |
| objective respond rate | 0.24 |
| Disease control rate | 0.56 |
| Overall survival | 0.34 |
| Progression-free survival | 0.89 |
| Any | 0.12 |
| Anemia | 0.87 |
| Diarrhea | 0.23 |
| Leukopenia | 0.18 |
| Nausea | 0.65 |
| Rash | 0.92 |
| Thrombocytopenia | 0.78 |


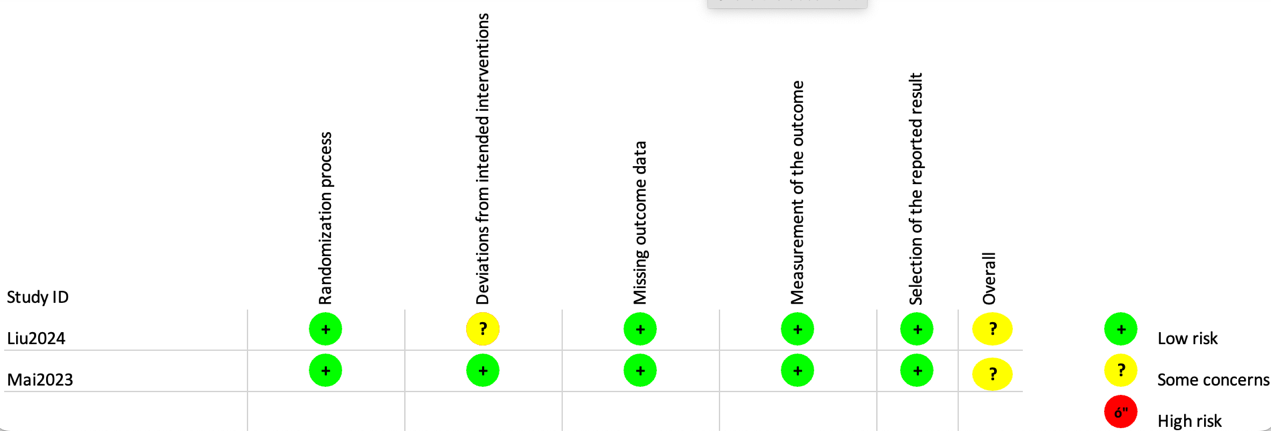


FigureS1 risk of bias


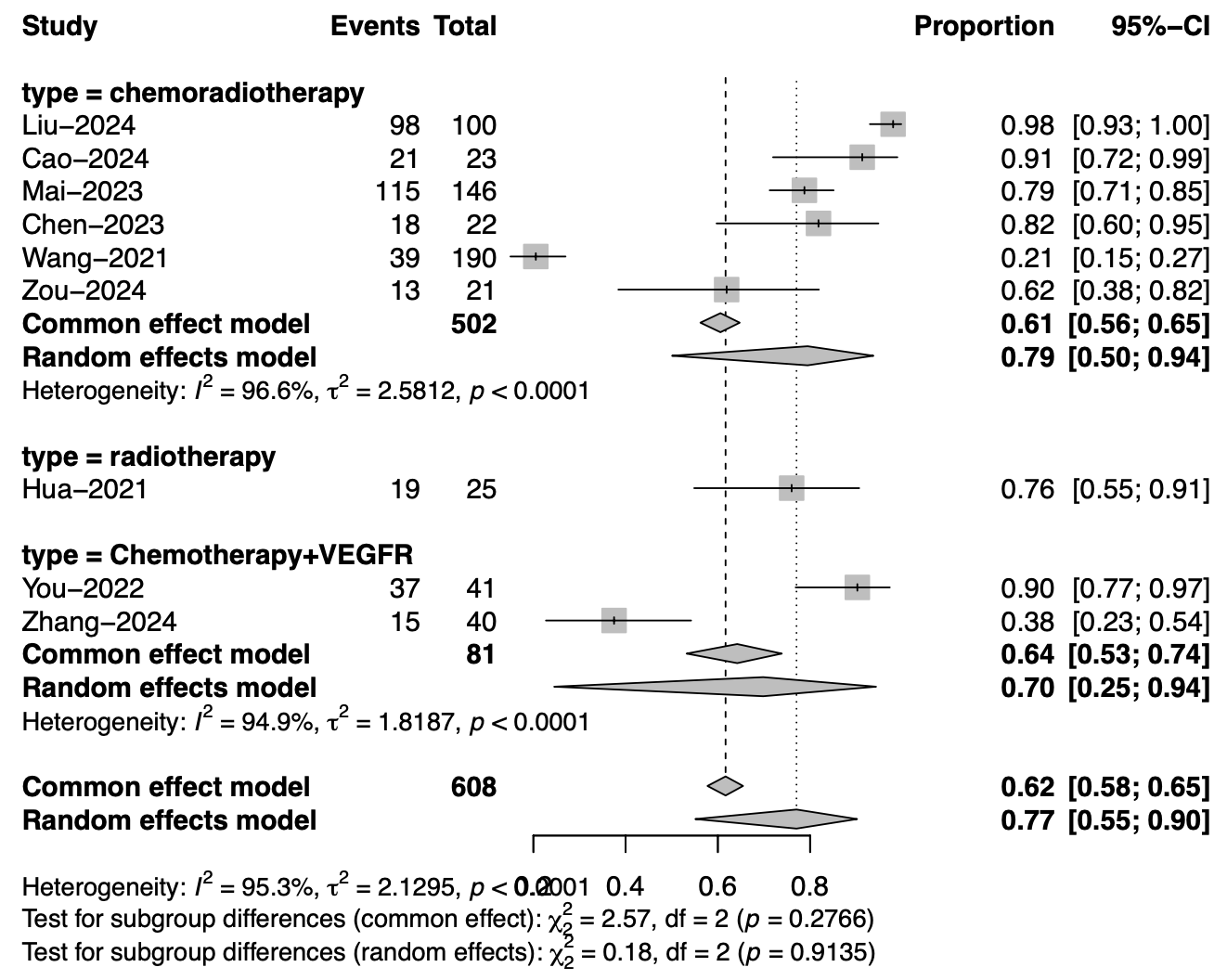


FigureS2 Forest plot of ORR combined treatment subgroup analysis


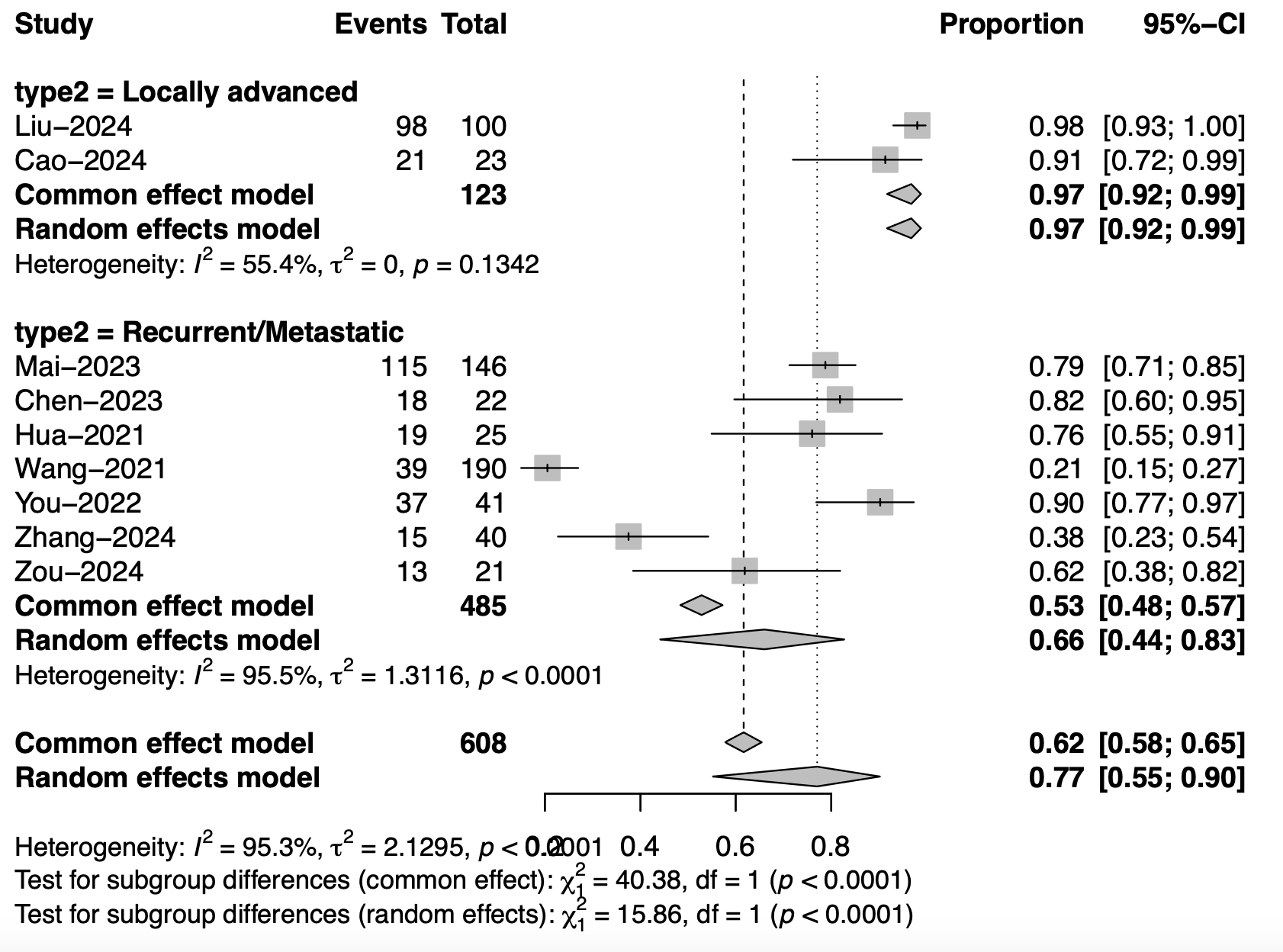


FigureS3 Forest plot of NPC type subgroup analysis by ORR


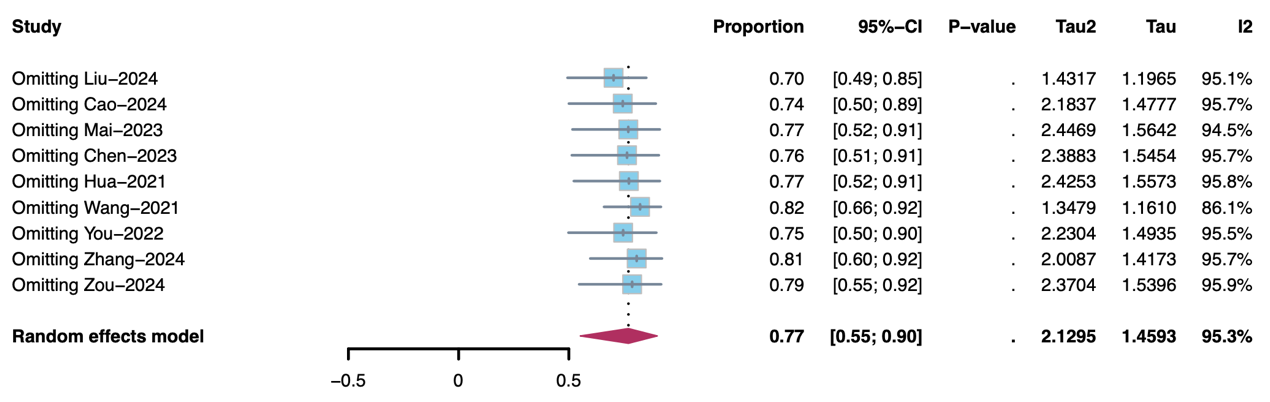


FigureS4 Forest plot of ORR sensitivity analysis


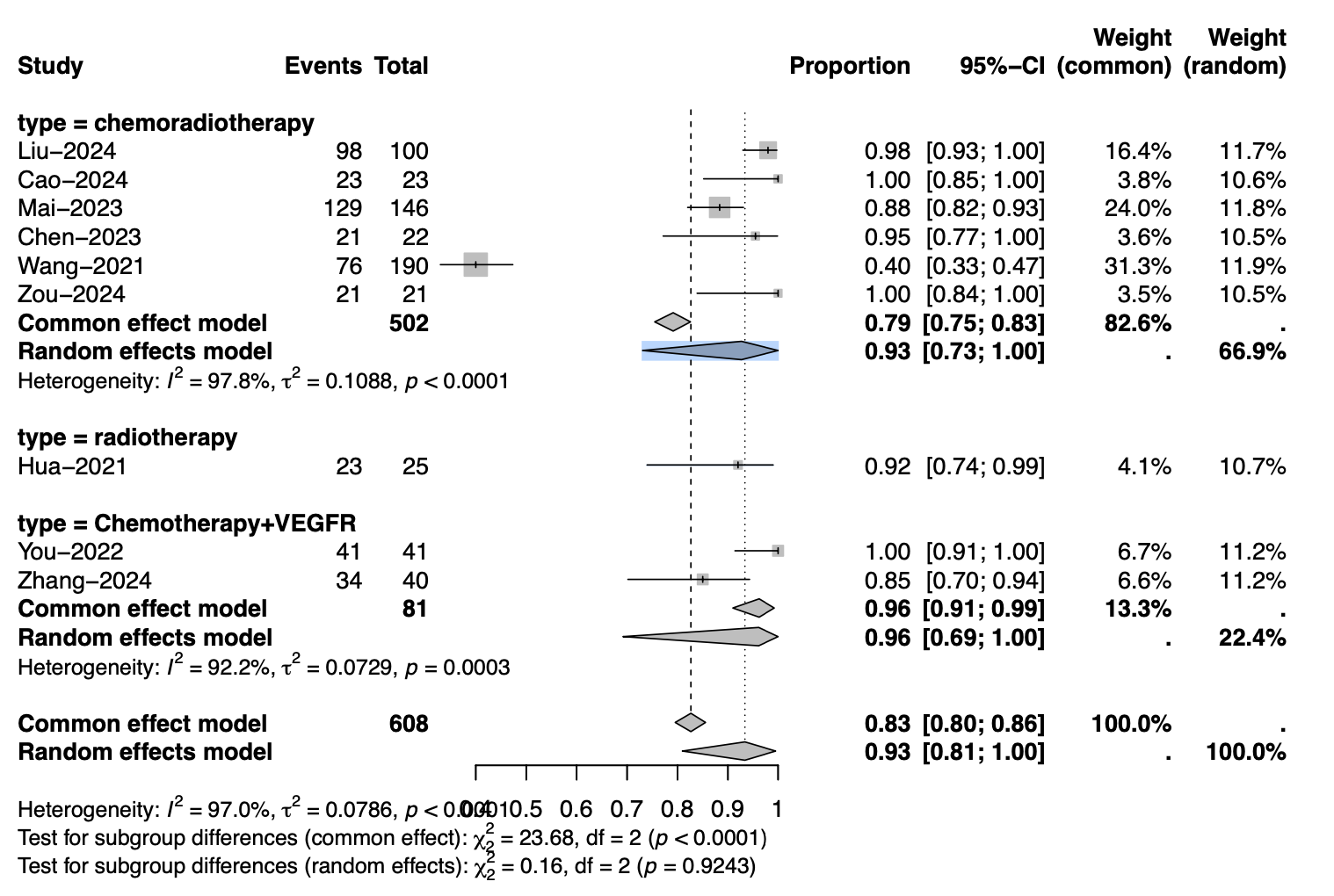


FigureS5 Forest plot of DCR combined treatment subgroup analysis


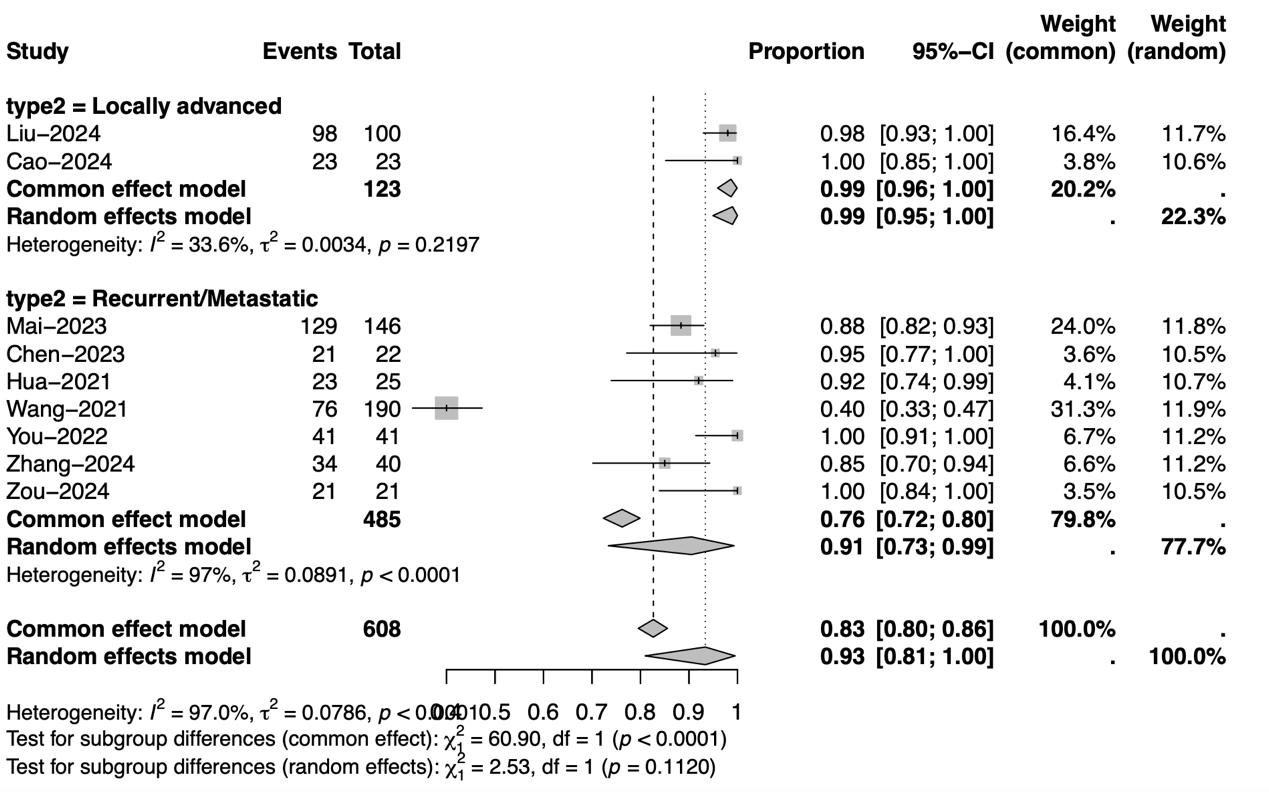


FigureS6 Forest plot of NPC type subgroup analysis by DCR


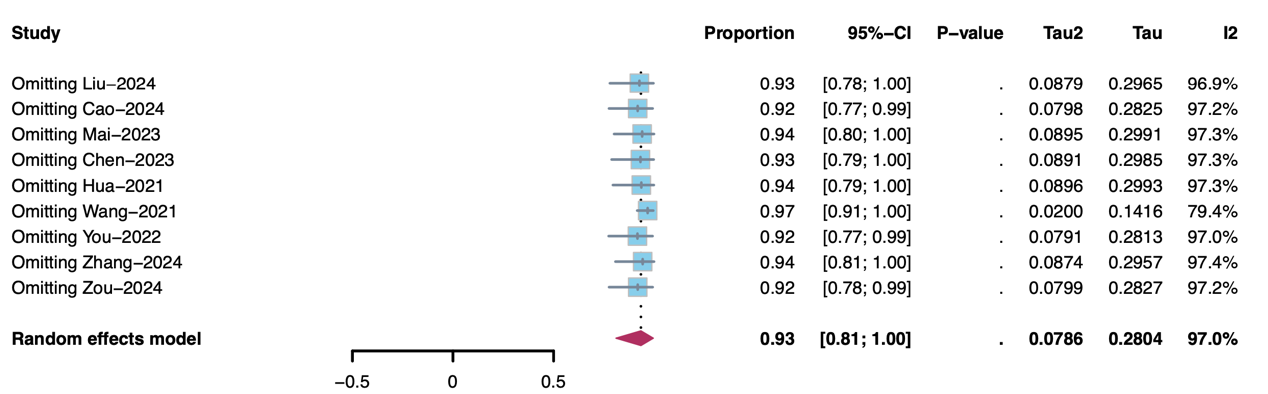


FigureS7 Forest plot of DCR sensitivity analysis


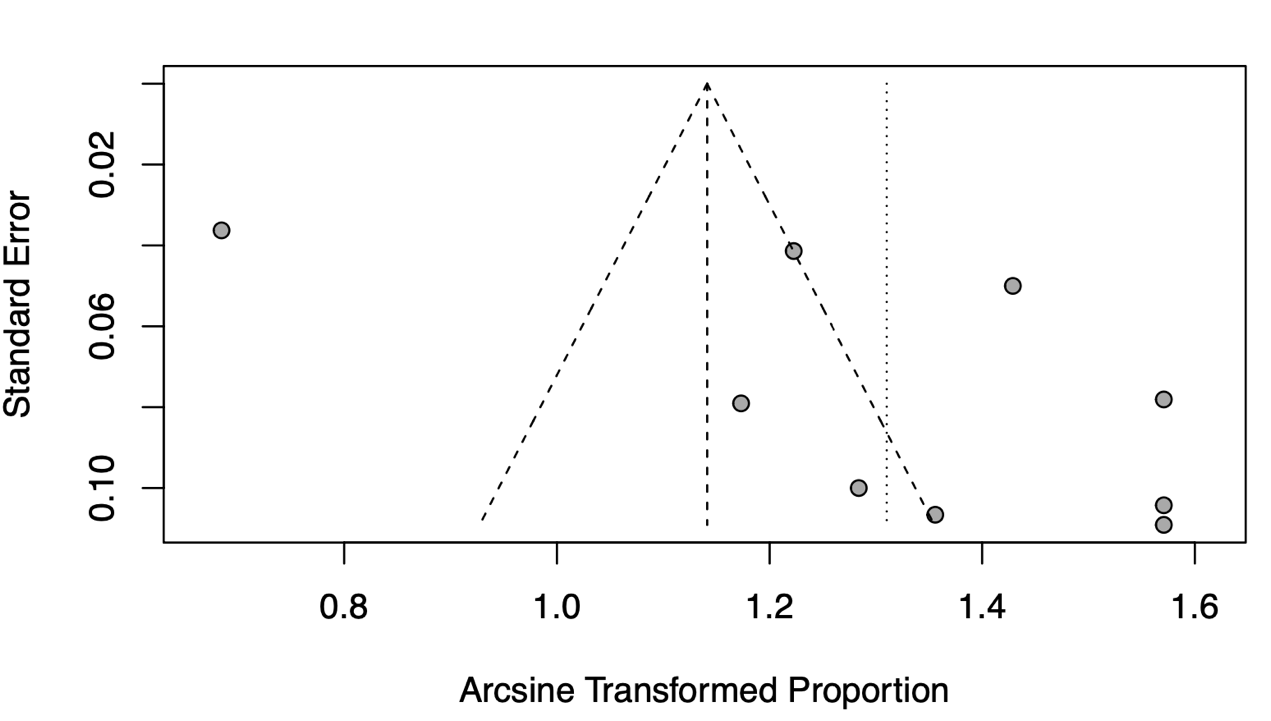


FigureS8 DCR funnel diagram


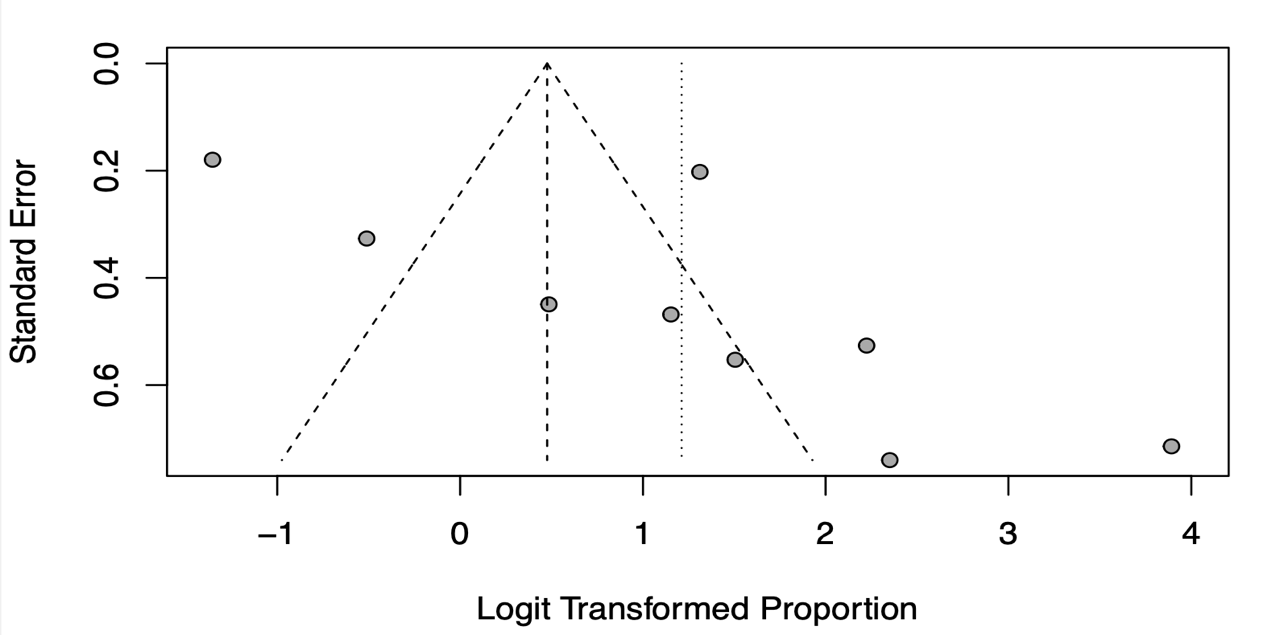


FigureS9 ORR funnel diagram


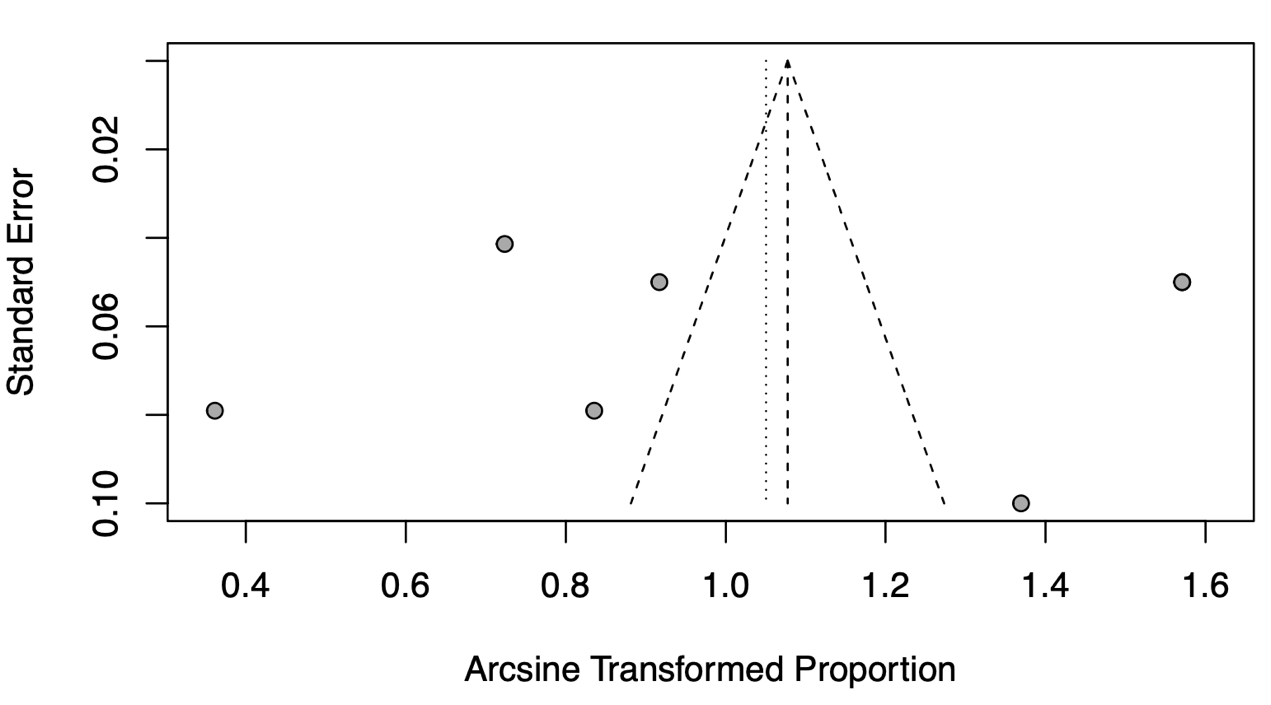


FigureS10 OS funnel diagram


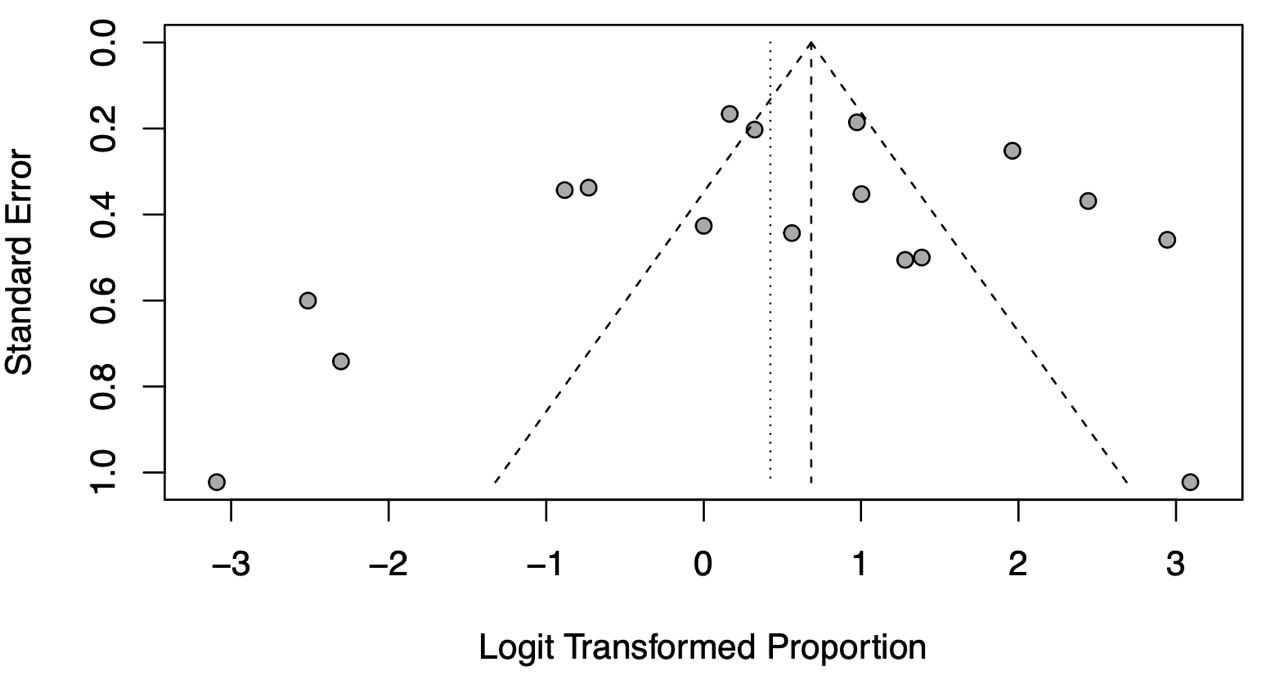


FigureS11 PFS funnel diagram
